# Effect of Tobacco Smoking on The Clinical, Histopathological, and Serological Manifestations of Sjögren’s Syndrome

**DOI:** 10.1371/journal.pone.0170249

**Published:** 2017-02-06

**Authors:** Donald U. Stone, Dustin Fife, Michael Brown, Keith E. Earley, Lida Radfar, C. Erick Kaufman, David M. Lewis, Nelson L. Rhodus, Barbara M. Segal, Daniel J. Wallace, Michael H. Weisman, Swamy Venuturupalli, Michael T. Brennan, Christopher J. Lessard, Courtney G. Montgomery, R. Hal Scofield, Kathy L. Sivils, Astrid Rasmussen

**Affiliations:** 1 Department of Ophthalmology, Johns Hopkins University, Baltimore, Maryland, United States of America; 2 King Khaled Eye Specialist Hospital, Riyadh, Kingdom of Saudi Arabia; 3 Arthritis and Clinical Immunology Research Program, Oklahoma Medical Research Foundation, Oklahoma City, Oklahoma, United States of America; 4 US Air Force 59th Medical Wing, Joint Base San Antonio-Lackland Air Force Base, San Antonio, Texas, United States of America; 5 Department of Oral Diagnosis and Radiology, University of Oklahoma College of Dentistry, Oklahoma City, Oklahoma, United States of America; 6 Department of Medicine, University of Oklahoma Health Sciences Center, Oklahoma City, Oklahoma, United States of America; 7 Department of Oral Pathology, University of Oklahoma College of Dentistry, Oklahoma City, Oklahoma, United States of America; 8 Department of Oral Surgery, University of Minnesota School of Dentistry, Minneapolis, Minnesota, United States of America; 9 Hennepin County Medical Center, Minneapolis, Minnesota, United States of America; 10 Division of Rheumatology, Cedars-Sinai Medical Center, Los Angeles, California, United States of America; 11 Department of Oral Medicine, Carolinas Medical Center, Charlotte, North Carolina, United States of America; 12 Department of Veterans Affairs Medical Center, Oklahoma City, Oklahoma, United States of America; Nippon Medical School, JAPAN

## Abstract

**Objectives:**

To assess the association of smoking habits with the clinical, serological, and histopathological manifestations of Sjögren’s syndrome (SS) and non-Sjögren’s sicca (non-SS sicca).

**Methods:**

Cross-sectional case-control study of 1288 patients with sicca symptoms (587 SS and 701 non-SS sicca) evaluated in a multi-disciplinary research clinic. Smoking patterns were obtained from questionnaire data and disease-related clinical and laboratory data were compared between current, past, ever, and never smokers.

**Results:**

Current smoking rates were 4.6% for SS patients compared to 14.1% in non-SS sicca (p = 5.17x10E-09), 18% in a local lupus cohort (p = 1.13x10E-14) and 16.8% in the community (p = 4.12x10E-15). Current smoking was protective against SS classification (OR 0.35, 95%CI 0.22–0.56, FDR q = 1.9E10-05), focal lymphocytic sialadenitis (OR 0.26, 95%CI 0.15–0.44, FDR q = 1.52x10E-06), focus score ≥1 (OR 0.22, 95%CI 0.13–0.39, FDR q = 1.43x10E-07), and anti-Ro/SSA(+) (OR 0.36, 95%CI 0.2–0.64, FDR q = 0.0009); ever smoking was protective against the same features and against anti-La/SSB(+) (OR 0.52, 95%CI 0.39–0.70, FDR q = 5.82x10E-05). Duration of smoking was inversely correlated with SS even after controlling for socioeconomic status, BMI, alcohol and caffeine consumption.

**Conclusions:**

Current tobacco smoking is negatively and independently associated with SS, protecting against disease-associated humoral and cellular autoimmunity. The overall smoking rate amongst SS patients is significantly lower than in matched populations and the effects of smoking are proportional to exposure duration.

In spite of the protective effects of tobacco on SS manifestations, it is associated with other serious comorbidities such as lung disease, cardiovascular risk and malignancy, and should thus be strongly discouraged in patients with sicca.

## Introduction

Tobacco use creates a tremendous burden on the health care system and is the largest non-communicable source of disease globally; annual tobacco-attributable deaths surpassed 5 million in 2010.[1, 2] Cigarette smoking has wide-ranging effects on the user depending on both extrinsic and intrinsic factors, with a well-described influence on oncogenesis, pulmonary function, vascular health, and immune response. [3–10] The mechanisms of disease may be as diverse as the contents of cigarette smoke; carbon monoxide, cyanide, nicotine, benzene, formaldehyde, methanol, ammonia, tar and nearly 4000 other chemicals identified in cigarette smoke.[11]

The dysregulation of immune and inflammatory responses caused by tobacco results in the association of smoking with various conditions that have inflammatory or autoimmune mechanisms as part of their pathophysiology.[9] Perhaps the best examples of associations of current tobacco smoking with increased incidence or severity of disease are with rheumatoid arthritis,[7, 8, 12–15] Crohn’s disease,[16] and multiple sclerosis.[17] The underlying mechanisms that have been postulated for this exacerbated inflammatory or autoimmune response include hypoxia and oxidative stress,[18] induction of pro-inflammatory cytokines and autoantibodies,[9] and epigenetic changes.[19]

However, despite leukocytosis and increases in markers of inflammation such as C-reactive protein in chronic smokers, there is impairment of some aspects of immune function and increased susceptibility to certain infections.[10] In some conditions, such as ulcerative colitis,[16] Behçet’s disease,[20] and sarcoidosis,[21] there is a negative correlation between smoking and disease activity or diagnosis. It is likely that specific gene-environment interactions for each disease are determinant in the effect that smoking exerts on the phenotype of inflammatory conditions.[8]

Second in prevalence among the rheumatic autoimmune diseases,[22] Sjögren’s syndrome (SS) is a chronic, systemic disease with a prototypical clinical presentation of xerostomia and xerophthalmia, associated with immune mediated dysfunction of the salivary and lacrimal glands.[23] Hallmarks of the autoimmune nature of SS are characteristic lymphocytic infiltrates of the salivary and lacrimal glands and the presence of circulating autoantibodies, mainly anti-Ro/SSA and anti-La/SSB,[24] as well as genetic association to genes involved in innate and adaptive immunity.[25] In addition to exocrine gland dysfunction, patients with SS have increased risk of cardiovascular disease,[26] interstitial lung disease and COPD,[27] and a ~20 times higher risk of B cell lymphoma.[23, 28] Few studies have evaluated the role of tobacco smoking in SS, a relevant consideration given the comorbidities of SS.

A decreased smoking rate in SS has previously been documented but detailed analysis of tobacco effects on SS manifestations is incomplete. Karabulut, et al.[29] reported a decreased prevalence of current smoking amongst SS patients in comparison to healthy controls but no association between smoking and autoantibodies, focus score, or extraglandular involvement.[29] Nilsson, et al. also found a significantly lower prevalence of smoking amongst SS patients assessed for COPD when compared to non-SS controls; interestingly, the non-smoking status of the SS patients was not protective against obstructive and possibly restrictive pulmonary disease.[27] Two European studies evaluating cardiovascular disease risk in SS reported low smoking rates,[26, 30] and an inverse correlation of current smoking with presence of anti-Ro/SSA and anti-La/SSB autoantibodies.[26] Finally, Manthorpe and colleagues[31] did not find differences in the smoking habits of SS and healthy individuals but determined that smoking at the time of minor salivary gland (MSG) lip biopsy was associated with lower risk of abnormal focus score and, to a lesser degree, with absence of circulating anti-Ro/SSA and anti-La/SSB autoantibodies.

Given the observed effect of tobacco smoking on the clinical manifestations of other autoimmune conditions and its association with conditions that disproportionately affect patients with SS, we sought to explore the effects of tobacco use on a carefully characterized population of patients with SS.

## Patients and Methods

### Study Participants and Ethical Considerations

The study subjects were 1288 voluntary participants in the Sjögren’s Research Clinics (SRC) at the Oklahoma Medical Research Foundation (n = 1007), the University of Minnesota (n = 214), Cedars-Sinai Medical Center (n = 57), and the Carolinas Medical Center (n = 10). Each institution’s Institutional Review Board approved all procedures and the participants provided two-tiered informed consent prior to entering the study. Initial verbal consent was obtained before the screening phone interview and the mailing of study paperwork and questionnaires; on the day of the clinic visit and before any procedure took place, additional written informed consent was obtained.

### Clinical Procedures

The protocols for patient recruitment, assessment, and data collection have been described previously[32] and include all the tests necessary for SS classification based on the American-European Consensus Group 2002 revised criteria[24].

Briefly, candidates for evaluation at the SRC were referred by health care providers or responded to public advertisement; they included patients already clinically diagnosed with SS and subjects with subjective dry eyes and dry mouth without a prior diagnosis of the disease. After explaining the objectives and methods of the study and obtaining verbal consent, trained personnel assessed the presence of ocular and oral symptoms using a validated telephone questionnaire and determined eligibility. Exclusion criteria followed the AECG classification[24] and included past head and neck irradiation, hepatitis C infection, acquired immunodeficiency syndrome, pre-existing lymphoma, sarcoidosis, and graft-versus-host disease as well as pregnancy and inability to provide informed consent. Additional exclusion criteria for this study were documented overlapping autoimmune conditions; thus, we only included in the analysis subjects that met criteria for primary SS or non-SS sicca. Use of anticholinergic medications was not considered as a factor for exclusion, but was documented.

The study subjects participated in multi-specialty clinical evaluation that included oral examination with measurement of whole unstimulated salivary flow (WUSF), minor salivary gland lip biopsy, and collection and storage of saliva. The ocular examination included slit lamp biomicroscopy, Schirmer’s I testing, calculation of the van Bijsterveld (vBS)[33] and ocular staining scores (OSS)[34] by staining of the cornea with fluorescein and the conjunctiva with lissamine green, and collection and storage of tears. Blood samples were taken for determination of relevant autoantibodies as well as extraction of DNA, RNA, and serum for other studies[32]. A physician with expertise in rheumatological diseases completed a detailed history and physical examination. If the patient reported a past or current diagnosis of another autoimmune condition, such as rheumatoid arthritis, mixed connective tissue disease, systemic sclerosis, myositis, primary biliary cirrhosis, multiple sclerosis, or systemic lupus erythematosus (SLE), classification criteria for these illnesses were specifically addressed by history, review of medical records, and directed diagnostic testing for the corresponding condition.

The histopathologic pattern of the salivary gland biopsy was determined according to the definitions by Daniels et al[35]:

1. Focal Lymphocytic Sialadenitis: presence of 1 or more dense aggregates of 50 or more lymphocytes (usually several hundred or more), usually located in perivascular or periductal locations. The foci are located adjacent to normal- appearing mucous acini in gland lobes or lobules lacking duct dilation or interstitial fibrosis and contain no more than a minority proportion of plasma cells. This diagnosis is assigned when these foci are the only inflammation present in a specimen, or the most prominent feature. Focus scores are then assigned by assessing the glandular area in each and calculating the number of lymphocytic foci present, per 4 mm2 of glandular area.

2. Non-specific Chronic Inflammation (Sialadenitis): scattered or focal infiltrates of lymphocytes, macrophages, and plasma cells that are not adjacent to normal-appearing acini and located in gland lobules that exhibit some combination of acinar atrophy, interstitial fibrosis, duct dilation, and luminal inspissated mucus.

3. Sclerosing Chronic Sialadenitis: An advanced stage of nonspecific chronic sialadenitis in which interstitial fibrosis, various patterns of chronic inflammation, and acinar atrophy predominate.

4. Within Normal Limits: diagnosed in minor salivary glands with normal-appearing architecture and scattered plasma cells, but without acinar atrophy and few if any lymphocytes.

5. Granulomatous Inflammation: Clusters of CD-68 positive macrophages, with or without occasional multinucleated giant cells and without necrosis.

Information about cigarette smoking was collected using a questionnaire mailed to the participants before attendance to the SRC. The questions about smoking addressed current or past smoking, age of onset, and duration of regular smoking. Questions about the number of cigarettes smoked on average were only added to the questionnaire in the last 18 months, so the data are not available for the majority of the study participants (S1 Appendix). Unanswered or incomplete questions were completed at the time of clinic participation with the help of a trained clinical coordinator. Smoking behaviors were defined as four categories: subjects reporting to be smokers at the time of study participation were classified as current smokers, those that reported a history of smoking but had quit were defined as past smokers while subjects that responded they had not been smokers were named never smokers. Ever smokers were defined as past plus current smokers. For each case, the time of the first diagnosis of SS or sicca syndrome (prior clinical diagnosis by referring physician or classification resulting from SRC evaluation) was considered in order to establish the temporal relation between smoking and disease. Multiple potential confounding variables were also assessed and detailed information can be found in the supplemental materials (S1 Appendix).

The comparison groups included a SLE cohort from the same institution with a similar geographic, age, and gender distribution,[36, 37] and community smoking prevalence data from the Centers for Disease Control (CDC),[38] also selected to match the age and gender distribution of the study cohort.

### Statistical Analysis

Count data were analyzed using Fisher’s exact, Pearson’s χ^2^, or Mann Whitney tests, as appropriate. Odds ratios (OR) and 95% confidence intervals (CI) were calculated using the maximum likelihood estimator (MLE) where applicable. The comparison of smoking prevalence in SS patients to the community data was done using a proportions (z) test. Comparisons of AECG criteria between current-past smokers and ever-never smokers were adjusted using the false discovery rate (FDR) method to correct for multiple testing. Path analysis, logistic regression models, and Random Forest algorithms[39] were used to estimate the marginal effect of smoking on SS after controlling for potential confounding variables (detailed path analysis and regression methods in S2 Appendix).

## Results

The cohort consisted of 1288 subjects: 596 participants entered the study with a clinical diagnosis of SS that was supported by pSS AECG classification at the SRC in 378 cases (63.4%); 651 had no prior clinical SS diagnosis but 190 (29.1%) met AECG criteria for pSS, while 41 (of which 19 [46%] were classified as pSS) were uncertain about the diagnostic impression of their referring physician. Thus, based on our comprehensive research evaluation at the SRC, 587 participants were classified as primary SS and 701 as non-SS sicca. The sociodemographic features of the participants are shown in Table 1. While SS and non-SS sicca participants had similar gender and ethnicity distributions, the latter were younger and less likely to be Asian (p = 9.39x10E-06 and p = 0.007, respectively), and more likely to be Native American (p = 0.03) than subjects classified as SS.

**Table 1:** Sociodemographic characteristics and smoking status of study participants with primary Sjögren’s syndrome and non-Sjögren’s sicca.

	Primary Sjögren’s Syndrome	Non-Sjögren’s sicca	*P value*
	n = 587	n = 701	
Age (median years [IQR])	56 [47–65]	52 [43–62]	**9.39E-06**^**a**^
Gender (female)	547 (93.2%)	643 (91.7%)	0.32^b^
Race^d^			
White	401 (68.3%)	442 (63.1%)	0.05^c^
Native American^e^	148 (25.2%)	216 (30.8%)	**0.03**^**c**^
African American	16 (2.7%)	25 (3.6%)	0.43^c^
Asian	14 (2.4%)	4 (0.01%)	**0.007**^**c**^
Ethnicity (non-Hispanic)	559 (95.2%)	680 (97%)	0.13^b^
Smoking status			
Current smoker	27 (4.6%)	99 (14.1%)	**5.17E-09**^**b**^
Past smoker	179 (30.5%)	230 (32.8%)	0.37^b^
Ever smoked	206 (35.1%)	329 (46.9%)	**9.20E-06**^**b**^
Never smoked	381 (64.9%)	372 (53.1%)	**0.0003**^**b**^
Smoking duration (median years [IQR])	15 [6.75–27]	19 [10–30]	**0.0013**^**a**^
Cessation (median years [IQR])	18 [7.5–30]	16 [5–28]	0.3789^a^
Education			
12 years or less	156 (26.6%)	192 (27.5%)	0.97^a^
College or equivalent	298 (50.8%)	353 (50.6%)	1.0^a^
Graduate/Professional or more	121 (20.6%)	150 (21.5%)	0.97^a^
Income (median USD [IQR])	50,486 [41,422–65,871]	49,560 [40,471–62,707]	0.08^a^
BMI (median [IQR])	27.95 [23.47-33-38]	27.62 [16.06–33.28]	0.98^a^

^a^ Mann-Whitney Test

^b^ χ^2^ Test

^c^ Fisher’s exact test

^d^Some data-points were not available; the percentages are calculated based on the subjects with data recorded.

^e^Native-American: Self-referred as either “Native-American” or “More than one race, one of which was Native-American”

BMI: Body Mass Index

We observed a significant negative association between both current and previous smoking status and SS classification. The current smoking rate among SS patients was only 4.6%, while 14.1% of non-SS sicca subjects were current smokers (p = 5.17x10E-09); these rates are in contrast to a SLE cohort from the same institution adjusted for age and gender,[36] in which the current smoking rate was 18% (*vs*. pSS p = 1.14x10E-14 and *vs*. non-SS p = 0.031), or to the national rate for women ages 45–64 of 16.8% (*vs*. pSS p = 4.12x10E-15 and *vs*. non-SS p = 0.168). [38] Approximately 35% percent of SS patients reported ever smoking, a significantly lower proportion than reported amongst the non-SS sicca controls (47%, p = 9.2x10E-06). The proportion of subjects who were past smokers but quit was not significantly different between SS patients and non-SS sicca participants (30% vs. 32%; p = 0.43), however, never smokers were more prevalent amongst SS (65% vs. 53%, p = 0.0003) (Table 1). When comparing current to past smokers, the current smokers had a significantly lower risk of being classified as SS than past smokers (OR 0.35, 95%CI 0.22–0.56, p = 3.0X10E-06, FDR q = 1.9X10E-05), and ever smokers had a lower risk than never smokers (OR 0.61, 95%CI 0.49–0.77, p = 9.2x10E-06, FDR q = 5.83E10-05).

To determine if there were confounding factors related to the presence of chronic dryness, we compared the overall smoking rate of our cohort (irrespective of SS classification) to community data obtained from the CDC National Health Interview Survey;[38] 9.8% of the sicca cohort smoked in comparison to the reported rate in Oklahoma of 17.2% (p = 2.27XE-12), or the national rate of 16.8% (p = 2.1X10E-11). These results support the notion that dryness, regardless of the underlying cause, is a deterrent for smoking.

Given the large proportion of Native American patients attending our clinic and their high smoking rates (national rate for Native American women is 32.5%),[38] we analyzed them separately and found that the overall current smoking rate of all Native American women in the cohort was significantly lower (11.8%) than expected (p = 3.1x10E-16). Amongst Native Americans, the risk of being classified as SS for current smokers vs. past smokers, and for ever smokers vs. never smokers was also significantly lower (OR = 0.34, 95%CI 0.13–0.67, p = 0.02 and OR = 0.51, 95%CI 0.33–0.78, p = 0.002, respectively).

Detailed clinical and laboratory features of all participants based on their smoking status and their descriptive statistics are shown in Table 2.

**Table 2:** Sociodemographic, clinical, and serological features of all study participants (irrespective of Sjögren’s classification) based on smoking status.

	Current Smoker	Past Smoker	Ever Smoked	Never Smoked
	n = 126	n = 409	n = 535	n = 753
Age (median years [IQR])	47 [41–55]	57 [48–66]	54 [45–63]	55 [44–63]
Gender (female)	112 (89%)	375 (92%)	487 (91%)	703 (93%)
Smoking duration (median years [IQR])	29.25 [20–36]	15 [6–27]	19 [8–30.5]	N/A
Sjögren’s Syndrome^a^	27 (21%)	179 (44%)	206 (39%)	381 (51%)
Subjective dry eyes	123 (98%)	392 (96%)	515 (96%)	720 (96%)
Subjective dry mouth	123 (98%)	397 (97%)	520 (92%)	733 (97%)
Schirmer’s (+)	40 (33%)^b^	157 (39%)^b^	197 (38%)^b^	284 (38%)^b^
Schirmer’s mm/5min (median [IQR])	10 [5–23.5]	8 [3–15]	8 [4–16]	8 [3–17]
WUSF (+)	60 (48%)^b^	226 (56%)^b^	286 (54%)^b^	409 (55%)^b^
WUSF mL/15min (median [IQR])	1.62 [0.7–3.13]	1.26 [0.41–2.9]	1.32 [0.45–3.0]	1.29 [0.42–2.86]
vBS (+)	51 (43%)^b^	169 (44%)^b^	220 (43%)^b^	327 (46%)
vB score (median [IQR])	3 [1–5]	3 [1–5]	3 [1–5]	3 [1–5]
Auto-antibodies				
Anti-Ro/SSA	15 (12%)	112 (27%)	127 (24%)	266 (35%)
Anti-La/SSB	12 (10%)	66 (16%)	78 (15%)	185 (25%)
ANA	86 (68%)	261 (64%)	347 (65%)	509 (68%)
Rheumatoid Factor	14 (11%)	83 (21%)	97 (18%)	164 (22%)
Histopathology				
Focus score ≥1	17 (14%)^b^	158 (42%)^b^	175 (35%)^b^	309 (46%)^b^
Focus score (mean±SD)	0.31±1.04	1.40±2.23	1.12±2.04	1.54±2.38
Focal lymphocytic sialadenitis	19 (17%)^b^	142 (44%)^b^	161 (37%)^b^	274 (46%)^b^
Non-specific chronic inflammation	79 (71%)^b^	152 (48%)^b^	231 (54%)^b^	283 (47%)^b^
Sclerosing chronic sialadenitis	3 (3%)^b^	10 (3%)^b^	13 (3%)^b^	12 (2%)^b^
Normal salivary gland	10 (9%)^b^	16 (5%)^b^	26 (6%)^b^	27 (5%)^b^
Serologic abnormalities				
Leukopenia	5 (4%)	24 (6%)	29 (5%)	69 (9%)
Hypergammaglobulinemia (IgG)	3 (2%)	52 (13%)	55 (10%)	103 (14%)
Low C3	2 (2%)	5 (1%)	7 (1%)	21 (3%)
Low C4	5 (4%)	22 (5%)	27 (5%)	34 (5%)
ESSDAI score (mean±SD)	2.91±4.18^b^	3.51±5.46^b^	3.37±5.18^b^	2.97±4.34^b^
ESSPRI score (mean±SD)	6.69±1.94^b^	6.84±1.65^b^	6.79±1.72^b^	6.25±2.21^b^

^a^Sjögren’s Syndrome: Subjects are classified as primary Sjögren’s Syndrome based on the AECG criteria.[24]

WUSF: Whole Unstimulated Salivary Flow; vBS: van Bijsterveld Score.

^b^ Some datapoints were not available; the percentages are calculated based on the subjects with data recorded.

The most significant differences were identified when comparing objective measures of autoimmunity (namely, autoantibody presence and focal lymphocytic sialadenitis) between smokers and non-smokers (Table 3). Current smokers had a lower risk for presence of antiRo/SSA (OR 0.36, 95%CI 0.20–0.64, p = 0.0004, FDR q = 0.002), hypergammaglobulinemia (OR 0.17, 95% CI 0.05–0.55, p = 0.0008, FDR q = 0.003) and, most notably, for MSG biopsy with a focus score ≥1 (OR 0.22, 95%CI 0.13–0.39, p = 7.54x10E-09, FDR q = 1.43x10E-07). These protective effects of smoking were consistent when comparing ever vs. never smoking. Ever smokers were less likely to be anti-Ro/SSA positive (OR 0.57, 95%CI 0.44–0.73, p = 4.47Ex10-06, FDR q = 5.82x10E-05), anti-La/SSB positive (OR 0.52, 95%CI 0.39–0.70, p = 6.13x10E-06, FDR q = 5.82x10E-05), and focus score ≥1 (OR 0.65, 95%CI 0.52–0.83, p = 0.0005 FDR q = 0.002). However, no significant differences were encountered in mean ESSDAI or ESSPRI scores or their individual domains between the different smoking behaviors (data not shown).

**Table 3:** Risk of classification as Sjögren’s syndrome and presence of associated disease criteria and histopathologic patterns based on smoking status.

	Current Smokers vs. Past Smokers			Ever Smokers vs. Never Smokers		
	OR (95%CI)	χ^2^ P value	FDR adjusted q value	OR (95%CI)	χ^2^ P value	FDR adjusted q value
**Clinical and Serological features**						
Sjögren’s Syndrome^a^	0.35 (0.22–0.56)	**3.0E10-06**	**1.9E10-05**	0.61 (0.49–0.77)	**9.2E10-06**	**5.83E-05**
Subjective dry eyes	1.8 (0.51–6.17)	0.36	0.53	1.18 (0.67–2.08)	0.57	0.722
Subjective dry mouth	1.24 (0.34–4.46)	0.74	0.823	0.95 (0.48–1.87)	0.87	0.87
Schirmer’s (+)	0.76 (0.49–1.15)	0.18	0.311	0.97 (0.77–1.22)	0.76	0.802
vBS (+)	0.95 (0.63–1.44)	0.82	0.823	0.92 (0.73–1.15)	0.46	0.624
WUSF (+)	0.75 (0.50–1.12)	0.16	0.304	0.96 (0.77–1.21)	0.75	0.802
Anti-Ro/SSA (+)	0.36 (0.20–0.64)	**0.0004**	**0.002**	0.57 (0.44–0.73)	**4.47E10-06**	**5.82E-05**
Anti-La/SSB (+)	0.55 (0.28–1.05)	0.07	0.166	0.52 (0.39–0.70)	**6.13E10-06**	**5.82E-05**
ANA (+)	1.22 (0.79–1.87)	0.36	0.526	0.88 (0.70–1.12)	0.31	0.491
Rheumatoid factor (+)	0.53 (0.30–0.93)	**0.02**	0.054	0.89 (0.68–1.17)	0.42	0.614
Focus score ≥1	0.22 (0.13–0.39)	**7.54E10-09**	**1.43E-07**	0.65 (0.52–0.83)	**0.0005**	**0.002**
Leukopenia (+)	0.66 (0.25–1.78)	0.41	0.556	0.57 (0.36–0.89)	**0.013**	**0.041**
Hypergammaglobulinemia (+)	0.17 (0.05–0.55)	**0.0008**	**0.003**	0.72 (0.51–1.02)	0.07	0.148
Low C3 (+)	1.30 (0.25–6.80)	0.75	0.823	0.46 (0.20–1.10)	0.07	0.148
Low C4 (+)	0.73 (0.27–1.96)	0.53	0.67	1.12 (0.67–1.89)	0.66	0.784
**Histopathologic patterns**						
Focal lymphocytic sialadenitis	0.26 (0.15–0.44)	**1.6E10-07**	**1.52E10-06**	0.70 (0.55–0.91)	**0.006**	**0.023**
Non-specific chronic inflammation	2.73 (1.71–4.35)	**8.59E10-06**	**4.08E-05**	1.88 (1.0–1.65)	0.05	0.136
Sclerosing chronic sialadenitis	0.86 (0.23–3.19)	0.823	0.823	1.52 (0.68–3.36)	0.301	0.491
Normal salivary gland	1.88 (.83–4.28)	0.126	0.266	1.35 (0.78–2.35)	0.289	0.491

^a^Sjögren’s Syndrome: Subjects are classified as primary Sjögren’s Syndrome based on the AECG criteria[24].

WUSF: Whole Unstimulated Salivary Flow; vBS: van Bijsterveld Score. Bolded items are statistically significant.

More detailed analysis of the effects of smoking on the histopathologic patterns observed in the MSG biopsy, revealed that current smokers had a lower frequency of focal lymphocytic sialadenitis (OR 0.26, 95%CI 0.15–0.44, p = 1.60x10E-07, FDR q = 1.52x10E-06) but significantly more non-specific chronic inflammation in the gland than past smokers (OR 2.73, 95%CI 1.71–4.35, p = 8.59x10E-06, FDR q = 4.08x10E-05). These differences were still present but to a lesser degree when comparing ever to never smokers (Table 3). The proportion of MSG biopsies with sclerosing chronic sialadenitis or normal salivary gland tissue was similar across all groups and so was the frequency of germinal center-like structures, atrophy, fibrosis, or fatty infiltration of the glands (data not shown).

Participants with confirmed SS were affected by their smoking status in similar ways to the effects identified in the complete cohort. Namely, current smokers had significantly lower rates of minor salivary gland lip biopsies with focus score ≥1 (p = 0.0009), focal lymphocytic sialadenitis (p = 0.0084), and of hypergammaglobulinemia (p = 0.048), while they had higher rates of non-specific chronic inflammation (p = 8.59x10E-06). Similarly, current smokers had low rates of positive biopsy results when compared to never smokers (p = 0.0095) and higher frequency of non-specific chronic inflammation (p = 0.027) (Table 4)

**Table 4:** Sociodemographic, clinical, and serological features of study participants with primary Sjögren’s Syndrome^a^ based on their smoking status.

	Current	Past	Ever	Never
	Smoker	Smoker	Smoked	Smoked
	n = 27	n = 179	n = 206	n = 381
Age (median years [IQR])	54 [44–58]	58 [49–66]	57 [49–66]	56 [46–65]
Gender (female)	23 (85%)	162 (91%)	185 (90%)	362 (95%)*
Smoking duration (median years [IQR])	27 [18–35]	14 [6–25]	17 [8–30]	N/A
Subjective dry eyes	27 (100%)	175 (98%)	202 (98%)	376 (99%)
Subjective dry mouth	27 (100%)	177 (99%)	204 (99%)	379 (99%)
Schirmer’s (+)	14 (54%)^§^	96 (55%)^§^	110 (55%)^§^	195 (53%)^§^
Schirmer’s mm/5min (median [IQR])	5 [3–12]	5 [3–10]	5 [3–10]	5 [2–12]
WUSF (+)	15 (58%)^§^	124 (70%)^§^	139 (68%)^§^	259 (69%)^§^
WUSF mL/15min (median [IQR])	1.33 [0.4–2.95]	0.92 [0.20–2.13]	0.96 [0.25–2.28]	0.88 [0.24–2.21]
vBS (+)	12 (50%)^§^	94 (58%)^§^	106 (57%)^§^	231 (65%)
vB score (median [IQR])	3.5 [2–6.75]	4 [1–7]	4 [1–7]	4 [2–7]
**Auto-antibodies**				
Anti-Ro/SSA	14 (52%)	104 (58%)	118 (57%)	237 (62%)
Anti-La/SSB	10 (37%)	58 (32%)	68 (33%)	163 (43%)
ANA	22 (82%)	141 (79%)	164 (80%)	317 (83%)
Rheumatoid Factor	7 (26%)	65 (36%)^§^	72 (35%)^§^	132 (35%)
**Histopathology**				
Focus score ≥1	15 (60%)^§^	137 (87%)^§^***	152 (83%)^§^**	266 (82%)^§^**
Focus score (mean±SD)	1.47±2.11	3.06±2.62**	2.85±2.61**	2.87±2.70**
Focal lymphocytic sialadenitis	11 (58%)^§^	105 (85%)^§^**	116 (82%)^§^*	216 (77%)^§^
Non-specific chronic inflammation	7 (37%)^§^	16 (13%)^§^****	23 (17%)^§^*	47 (18%)^§^*
Sclerosing chronic sialadenitis	0 (0%)^§^	2 (2%)^§^	2 (1%)^§^	6 (2%)^§^
Normal salivary gland	1 (5%)^§^	1 (1%)^§^	2 (1%)^§^	6 (2%)^§^
**Serologic abnormalities**				
Leukopenia	4 (15%)	16 (9%)	20 (10%)	49 (13%)
Hypergammaglobulinemia (IgG)	2 (7%)	45 (25%)*	47 (23%)	91 (24%)
Low C3	0 (0%)	3 (2%)	3 (2%)	12 (3%)
Low C4	2 (7%)	14 (8%)	16 (8%)	27 (7%)
ESSDAI score (mean±SD)	2.69±4.31^§^	3.33±5.20^§^	3.24±5.07^§^	3.02±4.11^§^
ESSPRI score (mean±SD)	6.75±0.96^§^	6.93±1.75^§^	6.90±1.66^§^	6.03±2.25^§^

^a^Sjögren’s Syndrome: Subjects are classified as primary Sjögren’s Syndrome based on the AECG criteria.

WUSF: Whole unstimulated salivary flow; vBS: van Bijsterveld Score.

^§^Some datapoints were not available; the percentages are calculated based on the subjects with data recorded.

* p<0.05

**p<0.005

***p<0.0005

****p<0.0001.

**Fig 1:**
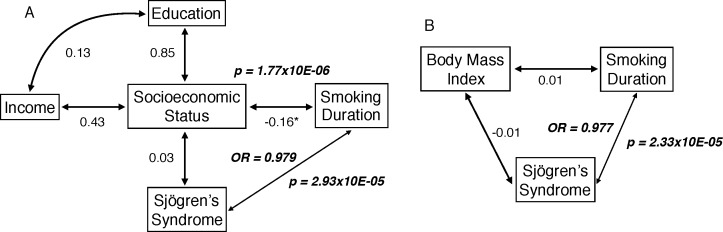
Path analysis model used to estimate the effect of smoking duration on SS and the role of potential confounding factors. The standardized path coefficients, significant odds ratios (OR) and p-values (in bold) are shown next to each path. A. Effect of smoking duration on SS, conditional on socioeconomic status; B. Model used to estimate the effect of smoking duration on SS, conditional on body mass index.

In an effort to control for confounding factors, we used logistic regression and two Path Analysis (PA) models (Fig 1, Table 5 and S2 Appendix) to determine whether the path coefficient from smoking duration to SS was significant after controlling for socioeconomic status (SES) and body mass index (BMI) as potential confounders. In both instances, smoking duration was independently protective (p = 2.93Ex10-05 and p = 2.33Ex10-05, respectively). In other words, the longer individuals smoke, the less likely they are to be diagnosed with SS, regardless of their SES or BMI. Similarly, controlling for caffeine and alcohol intake using a logistic regression model showed that smoking duration was independently protective from SS classification (p = 3.58Ex10-05). A similar analysis of the effect of time since smoking cessation on SS risk did not demonstrate a significant or independent effect.

**Table 5:** Logistic Regression models exploring the effect of covariates of smoking on the risk of classification as Sjögren’s Syndrome. The only significant predictor was smoking duration.

	Smoking Duration			
	Estimate	Std Error	z value	P value
**Model 1:**				
Smoking Duration	-0.02	0.01	-4.16	**3.14xE10-05**
Education	-0.03	0.02	-1.21	ns
Income	0.00	0.00	1.73	ns
**Model 2:**				
Smoking Duration	-0.02	0.01	-4.23	**2.33xE10-05**
BMI	0.00	0.01	0.11	ns
**Model 3:**				
Smoking Duration	-0.02	0.004	-4.09	**4.3xE10-05**
Alcohol intake	-0.06	0.06	-0.93	ns
Caffeinated beverages	-0.08	0.16	-0.47	ns

BMI: Body Mass Index.

Current smoking has a higher impact on SS classification than duration of smoking. The odds ratio for Sjögren’s syndrome classification in current smokers compared to never smokers is 0.27 (95% CI 0.17–0.42; p = 6.31x10E-10) and 0.35 (95% CI 0.22–0.56; p = 3.0x10E-06) in comparison to past smokers. The protective effect diminishes after cessation, so past smokers have an OR of 0.80 (95% CI 0.60–0.97; p = 0.026). In the case of smoking duration, the socioeconomic regression model shows that longer smoking duration results in an OR of Sjögren’s classification of 0.979 (95% CI 0.970–0.989; p = 2.93E-05) and the outcome of the BMI model shows and OR of 0.977 (95% CI 0.966–0.987; p = 2.33E-05). These effects are significantly different with non-overlapping 95%CI. (Fig 1 and S1 Appendix and S2 Appendix).

## Discussion

We explored the smoking behavior of a large cohort of patients with sicca syndrome and identified a strong protective effect of tobacco smoking against disease classification as primary SS and objective measures of autoimmunity. This effect persisted after correction for multiple testing and was consistently observed when comparing current to past smoking, and ever to never smoking. Exposure to tobacco smoke has been associated with a large number of deleterious effects on health. It is a well-known modulator of inflammatory and immune mechanisms and its use has been associated with an increase in disease risk, severity and flares of rheumatic diseases, in particular rheumatoid arthritis.[7, 8, 12–15] However, the precedent set by ulcerative colitis, Behçet’s disease, and aphthous stomatitis[16, 20] raises the possibility of a protective effect of smoking upon the development of some immune-mediated disorders. Few other studies have focused on the role of tobacco in Sjögren’s syndrome, which is a natural model to explore given that smoking has also been positively associated with oral and salivary gland diseases and symptoms of dry eyes.[40, 41]

The mechanism for the observed negative association between the classification of SS and tobacco use remains to be determined. Our results support the notion that the protective effect of smoking decreases after cessation since past smokers have intermediate rates of SS classification in comparison to current and never smokers; furthermore, current smoking was a significantly stronger determinant of SS risk than the total duration of smoking. The similar past smoking rates in the SS and non-SS sicca groups suggest that there is not an intrinsic difference between the groups regarding confounding factors that are associated with tobacco smoking. We performed a detailed analysis of known covariates of smoking, including SES, education, income, BMI, and alcohol and caffeine intake, and none of them were independent predictors of Sjögren’s classification. The overall current smoking rate of the cohort, irrespective of classification as SS or non-SS sicca, was significantly lower than expected based on the community smoking rates, even when adjusting for age, race, and gender. Given that the past smoking rates of the cohort as a whole are similar to the population rates, the low current smoking can only be explained by an increase in smoking cessation. A plausible explanation is that the mucosal drying and irritation caused by smoking are an effective deterrent in patients suffering from dryness.[42] This is further supported by the observed lower current smoking rate among non-SS sicca patients compared to the SLE and community comparison groups, despite similar past smoking rates.

The effects of tobacco on SS manifestations were confined to measures of objective autoimmunity. The proportion of study participants that reported subjective dry eyes and dry mouth was comparable between SS and non-SS sicca subjects, and also across all smoking behaviors. Furthermore, there were no significant differences in objective measures of lacrimal and salivary production between smokers and non-smokers. Previous studies have suggested that the main ocular surface effects of tobacco smoking are associated with abnormal tear breakup time rather than aqueous tear production;[40] this is likely the reason for the lack of association of smoking status with the Schirmer’s and ocular surface staining tests. Smoking has been associated with contradictory effects on salivary flow rate; in healthy patients, the short-term effect is increased saliva production that eventually leads to decreased salivary flow rate.[43] Neither our study nor earlier studies in SS patients have found significant effects of tobacco on whole unstimulated salivary flow.[31]

The most significant negative association of smoking was with a focus score ≥1 and with focal lymphocytic sialadenitis on the MSG biopsy; the mean focus score of smokers was also lower but did not reach statistical significance. These differences were observed when comparing current smoking to past, ever and/or never smokers. A previous study of SS patients had similar findings to our results, confirming lower MSG biopsy focus scores amongst current smokers albeit a detailed analysis of histopathologic features of the biopsies was not discussed.[31] Our results show not only that smokers are less likely to present with focal lymphocytic sialadenitis and focus scores ≥1, but also that they have significantly more non-specific chronic inflammation. These results suggest that the exposure to tobacco smoke protects against the formation of organized lymphocytic infiltrates in the salivary gland. The characteristic focal lymphocytic infiltrates in the exocrine glands of SS patients consist predominantly of activated CD4+ T lymphocytes (>75%) with B cells appearing in later stages of the disease.[44] The salivary gland pathology is mainly Th-1 mediated, particularly in cases with high focus scores, while activation of IL-17 secreting T-cells in the SS infiltrates promote the formation of germinal centers.[45, 46] Studies have shown that smoking causes a dose-dependent decrease in the number of CD4+ T cells, and that T cells from smokers proliferate poorly in response to T-cell mitogens.[47] Nicotine blocks pro-inflammatory cytokines with notable effects on IL-6, TH1, and IL-17-mediated responses.[48] Furthermore, chronic exposure to benzo[a]pyrene, an important component of cigarette smoke, results in decreases in the mass and cellularity of lymphoid tissues.[49]

When the exposure to cigarette smoke is sustained, a chronic inflammatory process is triggered that promotes enhanced microbial colonization and infection, persistence of apoptotic material and abnormal processing of cellular debris, with the potential to induce the architectural remodeling characteristic of chronic inflammation[50]. Several studies have shown that nicotine alters the morphology of salivary gland tissue. A study of parotid glands of rats exposed to nicotine in drinking water, demonstrated atrophy and swelling of acinary cells with an increase in the total intraacinar secretory granulae[51]; furthermore, smoke-exposed glands exhibit enlarged intercalated and striated ductal portions[52]. Additional cigarette-smoke effects are the dysregulation of innate immune responses in the oral cavity by modifying local TLR expression, distribution, and activation[53], thus promoting an environment permissive for chronic inflammation[54, 55]. So while tobacco has local inflammatory effects, its immunomodulatory mechanisms may underlie the reduced organized lymphocytic infiltration of the salivary gland observed in smoker SS patients and the preponderance on chronic non-specific inflammation.

In addition to the local exocrine gland pathology, patients with SS have alterations in the neuroendocrine system including the hypothalamus-pituitary-adrenal axis (HPA), the hypothalamic-pituitary-gonadal axis and the autonomic nervous system.[56] The HPA and autonomic nervous systems modulate local and systemic immune responses through nicotinic receptors in the brain, while non-neuronal nicotinic acetylcholine and cholinergic receptors in the periphery have been shown to modulate the Th17 response in CD4+ T lymphocytes[57]. Nicotine has been used experimentally in the management of ulcerative colitis, endometriosis, and sarcoidosis, but well-founded concerns about nicotine’s detrimental long-term and addictive effects have limited its therapeutic applications[10]. The exploration of alternative and selective nicotinic receptor agonists may lead to novel therapeutic options for the treatment of inflammatory and autoimmune disorders, including Sjögren’s Syndrome.

The presence of autoantibodies against Ro/SSA and La/SSB are characteristic of SS; the overall rate of anti-Ro/SSA(+) and anti-La/SSB(+) in our cohort is 31% and 20%, respectively (61% and 40% in SS patients). This is in contrast with the significantly lower 12% anti-Ro/SSA(+) and 10% anti-La/SSB amongst current smokers. We did not observe any significant differences in the proportion of positive ANA and rheumatoid factor (RF). As is the case with salivary flow rates, the relationship between smoking and autoantibodies has historically been contradictory (Table 6). Long-term smoking significantly reduces serum levels of immunoglobulins in humans but may increase the levels of some autoantibodies, in particular ANA.[10] Decreased prevalence of anti-Ro/SSA antibodies amongst patients with SS had been reported by Bartoloni et al[26] and of both antiRo/SSA and anti-La/SSB by Manthorpe et al.;[31] however, the study by Karabulut only found a positive association of current smoking with elevated ANA[29]. The results are also inconsistent in other autoimmune disorders. Some studies of SLE patients demonstrated an increased expression of anti-dsDNA antibodies amongst smokers [58] while other investigators did not find a clear association between smoking status and individual autoantibodies.[37] In the case of RA patients, smoking has been associated with increased ANA positivity in men and RF in women.[59, 60] These seemingly opposite results suggest that the role of smoking on pathogenic autoantibodies differs from one autoimmune disease to another and may be a good example of gene-environment interaction.

**Table 6:** Studies on the association between smoking and Sjögren’s Syndrome

Author	Study Population	Study Objective	Effects of tobacco smoking	Ref
Stone et al	587 pSS^a^	Prevalence of smoking in SS and association of smoking habits with clinical features and risk of SS	Lower prevalence of pSS	Current study
	701 non-SS sicca USA single center		Lower frequency of FS≥1	
	1242 SLE		Lower prevalence of focal lymphocytic sialadenitis	
	981 unaffected first-degree relatives and 946 healthy controls from USA LFRR registry		Higher prevalence of non-specific chronic inflammation of minor salivary glands	
			Lower frequency of anti-Ro/SSA +	
			Lower frequency of anti-La/SSB +	
			Inverse correlation between smoking duration and SS risk	
Bartoloni et al	788 pSS^a^ from 5 Italian centers	Compare prevalence of traditional cardiovascular disease risk factors and over disease	Lower frequency of anti-Ro/SSA +	(26)
	4774 control females from Registry of Italian General Population		Lower frequency of anti-La/SSB +	
			Lower prevalence of pSS	
Nilsson et al	51 pSS^a^ Malmö, Sweden	Prevalence of COPD with pSS and its association with cigarette smoking	Increased risk of COPD in pSS even amongst non-smokers	(27)
	186 control females from Uppsala general health survey			
Manthorpe et al	355 pSS^a^ Malmö, Sweden	Correlation of smoking habits with focus score in lower lip biopsies, serum antibodies and IgG	Lower frequency of FS≥1	(31)
	35 stomatitis sicca		Lower frequency of anti-Ro/SSA +	
	3700 age and sex matched controls from general population		Lower frequency of anti-La/SSB +	
Karabulut et al	207 pSS^a^ Turkey	Frequency of smoking in pSS and correlation with autoantibodies and extraglandular manifestations	Lower current smoking in pSS	(29)
	602 gender matched healthy controls		Higher past or never smoking in pSS	
			Higher frequency of ANA +	

^a^Subjects are classified as primary Sjögren’s Syndrome based on the AECG criteria^31^

The effects of tobacco have been shown to be proportional to lifetime exposure, so that former smokers have intermediate levels of disease features associated with tobacco in comparison to current and never smokers.[3] Measures of exposure include smoking duration, time since cessation, and intensity of smoking. Unfortunately, the smoking data collected in our study does not allow us to measure the intensity of smoking, measured as pack-per-year, so the dose-response analysis was limited to duration of exposure rather than the dosage of tar and nicotine as potential factors influencing our results. Similarly, we did not take into account other tobacco delivery systems that may influence the doses of each chemical; it has been reported that high levels of tar and nicotine induce immunosuppression faster and possibly for longer time than cigarette smoke with lower levels.[10] Animal models treated with nicotine were immunosuppressed for several weeks after cessation; similarly, patients with ulcerative colitis remained relapse free for several months after the end of nicotine treatment[10]. The effect of cessation was not replicated in our study, possibly because the vast majority of ever smokers discontinued smoking long before being diagnosed with either SS or non-SS. However, duration of smoking was significantly and inversely correlated with SS classification; to rule out that this was a spurious effect, we controlled for potential confounding factors by detailed multivariate analyses[39] and confirmed that smoking duration was an independent effect.

While we are aware of the significant limitations of a cross-sectional observational study like ours, we are encouraged by the solidity of our preliminary findings. Any probabilistic causal association between tobacco and SS would have to survive scrutiny for temporal relationship, biological plausibility, consistency with other independent studies, elimination of confounding factors, dose-response relationship, strength of association and cessation of effects[61, 62]. Our data support most of these factors, with the underlying biological mechanisms being the least explored but most attractive area of future exploration. Designing a prospective, longitudinal study of those at high risk for developing SS may shed light on the nature of the negative association with tobacco smoking and pinpointing the chemicals within the tobacco smoke responsible for the protective effects could open new avenues for understanding physiopathogenic pathways and potential therapeutic targets.

However, it is of the upmost importance to highlight that given the overwhelming negative effect of tobacco use on health, smoking cessation should be recommended to patients with Sjögren’s syndrome who are already at higher risk for malignancy, pulmonary and cardiovascular disease.

## Supporting Information

S1 Appendix Smoking questionnaire. (DOCX)

S2 Appendix Path analysis, logistic regression, and Random Forest Modeling. (DOCX)
