# Supplementary material for: Effect of Tobacco Smoking on The Clinical, Histopathological, and Serological Manifestations of Sjögren’s Syndrome
Source: PLoS One. 2017 Feb 6;12(2):e0170249. doi: 10.1371/journal.pone.0170249 (PMC5293551; doi:10.1371/journal.pone.0170249)
Supplement: S1 Appendix — (DOCX) [file pone.0170249.s001.docx]

**S1 Appendix**

**Tobacco use questionnaire (2006-2015):**

1. Have you ever smoked cigarettes?
2. How old were you when you started smoking?
3. How long did you (or have you) smoked?
4. Do you smoke cigarettes now?
5. Have you ever tried to quit?
6. If you quit entirely, how old were you when you quit?

**Tobacco use questionnaire (2015-2016):**

1. Have you smoked at least one hundred cigarettes during your lifetime?
2. If YES, during the period when you were smoking the most, how many cigarettes did you usually smoke? per day _______ OR per week _______
3. Have you ever smoked cigarettes regularly?
4. How old were you when you started smoking?
5. How long did you (or have you) smoked?
6. Do you smoke cigarettes now?

❑Every day

❑ Some days

❑ Not at all, I quit when I was _______ years old

1. On average, about how many cigarettes a day do you now smoke?
2. During the past 12 months, how many times have you stopped smoking for one day or longer because you were trying to quit smoking cigarettes for good?
3. Thinking back to the LAST TIME you QUIT or tried to QUIT smoking in the past 12 months, did you use ANY of the following products?
   1. A nicotine patch
   2. A nicotine gum or nicotine lozenge
   3. A nicotine nasal spray or nicotine inhaler
   4. A prescription pill called Chantix or Varenicline?
   5. A prescription pill called Zyban, Bupropion, or Wellbutrin?
4. Have you ever used any other forms of tobacco regularly?
5. If YES, please indicate which one(s):

❑ cigars or little cigars ❑ pipe ❑ smokeless/chewing tobacco ❑ hookah

1. If YES, are you currently using any of these other forms of tobacco on some days or everyday?

❑ cigars or little cigars ❑ pipe ❑ smokeless/chewing tobacco ❑ hookah

1. Have you ever used an e-cigarette or nicotine vapor device, even one or two times?
2. Do you now use an electronic cigarette or vapor device every day, some days, or not at all?
