# Supplementary material for: Effect of Tobacco Smoking on The Clinical, Histopathological, and Serological Manifestations of Sjögren’s Syndrome
Source: PLoS One. 2017 Feb 6;12(2):e0170249. doi: 10.1371/journal.pone.0170249 (PMC5293551; doi:10.1371/journal.pone.0170249)
Supplement: S2 Appendix — (DOCX) [file pone.0170249.s002.docx]

**S2 Appendix**

**Conditional Analysis**

**Background**

We have shown relationships between Sjögren’s syndrome and smoking indicating that smoking is protective against Sjögren’s. The purpose of this study is to investigate whether this relationship is spurious by controlling for other potentially confounding factors.

For the majority of the paper, we analyzed the variable “Smoking Duration,” and this value was obtained by averaging the computed smoking duration value and the self-reported smoking value. The smoking duration of never smokers was recorded as a smoking duration of zero.

**Confounding variables**

1. Do you drink alcohol? ❑ NO

❑ RARELY (less than once/month)

❑ SOMETIMES (between 1-10 times/month)

❑ MOST DAYS (more than 10 times/month) ❑ EVERYDAY

1. Do you drink caffeinated beverages?
2. If YES, how many cups or glasses do you drink ***per day***?

**Coding of variables:**

Alcohol: no = 0, rarely = 1, sometimes = 2, most days = 3, everyday = 4

Caffeine: no = 0, yes = 1

Body Mass Index (BMI): numeric value resulting from metric-derived measures

Education: education: college = 14.5, graduate school = 19, grade school = 4

Income: Median income in US dollars based on residential address zip code. [Data derived from the United States Census 2010 and American Community Survey and summarized by The University of Michigan Population Studies Center] [38]

**Socioeconomic status as a moderator of smoking**

**A. Path Analysis**

To determine whether socioeconomic status (SES), which is a function of education and income, explains the relationship between smoking and Sjögren’s, we performed a path analysis (PA) model^1^ using the model specified in Figure 1A. Residual variances of endogenous variables were freely estimated and the residual variance of Socioeconomic Status was fixed to zero. The values shown were estimated from the PA and the values with an asterisk were significant (p < 0.05). Notice that, even after controlling for SES, smoking is still a significant negative predictor of SS (z = −4.179, p = 2.93x10E−05); thus, subjects who smoke longer are less likely to develop SS.^^[[1]](#footnote-1)^^

**B. Logistic Regression**

To provide further evidence that the relationship between SS and smoking is *not* a function of SES, we also performed a logistic regression. For the logistic regression analysis, education and income were added as separate variables, rather than aggregating into a latent SES variable them as we did in the PA model. Table 4 in the manuscript shows these results. Note how the only significant predictor of SS is smoking duration (p = 3*.*14x10E-05), providing additional evidence that SES (i.e., income and education) is not responsible for the correlation between smoking and SS.

|  | Estimate | Std. Error | z value | p |
| --- | --- | --- | --- | --- |
| Smoking Duration | -0.02 | 0.01 | -4.16 | 0.00 |
| Education | -0.03 | 0.02 | -1.21 | 0.22 |
| Income | 0.00 | 0.00 | 1.73 | 0.08 |

**Body Mass Index as a moderator of smoking**

**A. Path Analysis**

As before, we investigated whether another variable was responsible for the correlation between smoking and SS. This time, however, we conditioned on body mass index (BMI). Figure 1B shows the model that was input into the PA machinery. Smoking duration was a significant predictor of Sjögren’s, independent of BMI. Only the path from smoking to SS was significant (p = 2*.*33x10E-05).

**B. Logistic Regression**

As before, we also included a logistic regression model and the results concur with the PA results, confirming that BMI is not a modifier of the protective effect of smoking on SS (Table 4 of the manuscript).

|  | Estimate | Std. Error | z value | Pr(*>\|*z*\|*) |
| --- | --- | --- | --- | --- |
| Smoking Duration | -0.02 | 0.01 | -4.16 | 0.00 |
| BMI | 0.00 | 0.01 | 0.11 | 0.91 |

**Alcohol and Caffeine intake as moderators of smoking**

**A. Logistic Regression**

The conditional analysis for the alcohol and caffeine variables was performed exclusively by logistic regression because the PA model would yield identical (or nearly identical) results to the logistic regression analysis since none of the predictor variables are endogenous. Again, the only significant variable in this model was smoking duration (p = 3.58x10E-05).

**Random Forest Algorithm**

There is a possibility that some of the variables noted above (SES, BMI, Alcohol, Caffeine) may interact in such a way that the relationship between smoking and SS vanishes. None of the above analyses are sensitive to these potential interactions. Consequently, we also performed a Random Forest (RF) analysis. RF natively detects interactions with the additional benefit that it cross-validates well and avoids over-fitting data.

We used a variable selection algorithm [39] and computed variable importance of each variable. The algorithm found only smoking duration to be a significant predictor (with an OOB of 0.45 and a variable importance of 0.005). Again, this demonstrates that smoking duration is predictive of SS, independent of SES (income/education), BMI, alcohol or caffeine consumption.

**References**

38. Jamal A, Homa DM, O'Connor E, Babb SD, Caraballo RS, Singh T, et al. Current cigarette smoking among adults - United States, 2005-2014. MMWR Morb Mortal Wkly Rep. 2015;64(44):1233-40. doi: 10.15585/mmwr.mm6444a2. PubMed PMID: 26562061.

39. Genuer R, Poggi JM, Tuleau-Malot C. Variable selection using random forests. Pattern Recogn Lett. 2010;31(14):2225-36. doi: 10.1016/j.patrec.2010.03.014. PubMed PMID: WOS:000282384500018.

1. Technically, this is a structural equation model (SEM) since latent variables are used in addition to observed variables. However, since the same machinery was used for the remainder of this report, and to avoid confusion, we call them all Path Analysis models. [↑](#footnote-ref-1)
